# Subtype specific immune-metabolic reprogramming in preeclampsia revealed by multiomics and serum biomarkers

**DOI:** 10.1038/s41440-025-02504-5

**Published:** 2025-12-19

**Authors:** Yixuan Chen, Linlin Wu, Dongni Huang, Xiaoxia Wu, Kan Liu, Bo Sun, Jinying Yang, Baozhen Zhang, Zijun Ouyang, Cuilian Zhang, Lunbo Tan, Jianmin Niu

**Affiliations:** 1 Department of Reproductive Medical Center, Henan Provincial People’s Hospital, People’s Hospital of Zhengzhou University, Henan Provincial People’s Hospital of Henan University, Zhengzhou, Henan China; 2 Department of Obstetrics, The Eight Affiliated Hospital, Sun Yat-Sen University, Shenzhen, China; 3 Women and Children’s Hospital of Chongqing Medical University, Chongqing, China; 4 Department of Obstetrics, Shenzhen Maternity & Child Healthcare Hospital, Guangdong, China; 5 Department of Obstetrics, Henan Provincial People’s Hospital, People’s Hospital of Zhengzhou University, Henan Provincial People’s Hospital of Henan University, Zhengzhou, Henan China; 6 Department of Obstetrics, Shenzhen Baoan Women’s and Children’s Hospital, Shenzhen, China; 7 Department of Obstetrics, Longgang Maternity and Child Clinical Institute, Shenzhen, China; 8 School of Food and Drug, Shenzhen Polytechnic University, Shenzhen, China

**Keywords:** Preeclampsia, Hofbauer cells, Immune-metabolic remodeling, Morning hypertension, Digital hypertension

## Abstract

Preeclampsia comprises early-onset (EOPE) and late-onset (LOPE) subtypes with distinct etiologies, placental pathology, and severity, but cellular/metabolic drivers and early biomarkers remain unclear. We integrated placental single-cell RNA-seq, spatial transcriptomics, and spatial metabolomics from EOPE, LOPE, and matched controls, and performed maternal serum metabolomics in a prospective cohort of 199 pregnancies. The scRNA-seq identified 14 cell types; Hofbauer cells and trophoblasts resolved into 7 and 3 subclusters. EOPE placentas showed increased macrophages and extravillous trophoblasts, reduced oxygen-transporting Hofbauer subtypes (HB_1, HB_6), and trophoblasts with heightened HIF-1, VEGF, and IGF signaling. LOPE preserved cellular composition but exhibited stronger inflammatory transcriptional programs. Spatial analyses indicated disrupted oxygen transport in EOPE and perturbed interferon-γ signaling and exosome secretion in LOPE. Metabolically, trophoblasts and Hofbauer cells displayed subtype-specific lipid-transport defects and mitochondrial dysfunction. Three early-pregnancy serum metabolites—phosphatidylcholine PC(22:5/0:0), 3-hydroxybutyric acid, and L-allothreonine—robustly predicted EOPE (AUC > 0.85). This study delineates preeclampsia as a spectrum of placental immune-metabolic disorders. Hofbauer cells and trophoblasts undergo subtype-specific transcriptional and metabolic remodeling in EOPE vs LOPE. Multi-omics-guided, noninvasive biomarkers enable early EOPE risk prediction, informing timely detection and intervention.

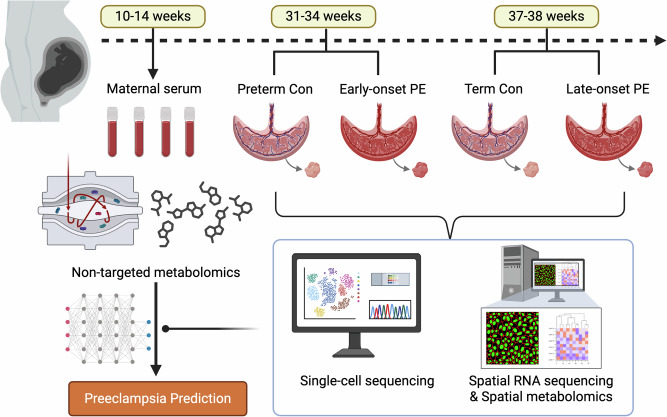

## Introduction

Preeclampsia is a complex hypertensive disorder of pregnancy, defined by new-onset hypertension and proteinuria after 20 weeks of gestation, affecting approximately 8% of pregnancies worldwide [1, 2]. This condition poses significant risks to both maternal and fetal health, with potential complications including placental abruption, maternal organ dysfunction, and adverse neonatal outcomes [1].

Traditionally classified into early-onset (<34 weeks) and late-onset (≥34 weeks) subtypes, preeclampsia is increasingly recognized as a syndrome with distinct etiological and pathological origins [3]. Early-onset preeclampsia (EOPE) is primarily associated with placental insufficiency and abnormal trophoblast invasion, whereas late-onset preeclampsia (LOPE) is more often linked to maternal cardiovascular or metabolic dysfunction [4]. These distinctions underscore the need for subtype-specific investigations to unravel the unique molecular and cellular mechanisms underlying these conditions.

Emerging evidence highlights the critical role of placental pathology in preeclampsia, particularly the altered behaviors of trophoblasts, immune cell populations, and placental vascularization [1, 2, 4, 5]. Hofbauer cells, the resident macrophages of the placenta, and trophoblasts, essential for implantation and nutrient exchange, are key players in maintaining placental homeostasis. Their dysfunction, characterized by pro-inflammatory phenotypes and impaired adaptation to hypoxia, has been implicated in preeclampsia pathogenesis. Recent advancements in single-cell technologies, such as single-cell RNA sequencing (scRNA-seq) and spatial transcriptomics, have revolutionized our ability to explore the cellular heterogeneity and functional states of placental tissues [6]. These tools enable researchers to identify altered cellular interactions and signaling pathways, offering critical insights into disease mechanisms.

In this study, we applied an integrative multi-omic strategy, including scRNA-seq, spatial transcriptomics, and spatial metabolomics on placental tissues from both early- and late-onset preeclampsia and gestational age-matched controls. Additionally, we performed untargeted serum metabolomics in a prospective pregnancy cohort to identify non-invasive early biomarkers. Through this multi-layered approach, we aimed to elucidate cell type-specific immune-metabolic dysfunctions underlying different preeclampsia subtypes and to explore the translational potential of early serum markers for disease prediction.

Point of view

- Clinical relevance**:** Multi-omics profiling reveals distinct immune-metabolic dysfunction in EOPE and LOPE, and supports a three-metabolite serum panel for early EOPE prediction.
- Future direction**:** Prospective multicenter studies with functional validation are required to confirm lipid-transport and oxygen-response pathways and refine biomarker performance.
- Consideration for the Asian population: The identified cellular signatures and serum metabolites were derived from an entirely Asian cohort and may reflect region-specific biological characteristics and clinical risk profiles.

## Materials and methods

### Ethics approval and consent to participate

All participants were recruited from Shenzhen Maternity and Child Health Care Hospital after providing written informed consent. The study protocol was approved by the Ethics Committee of Shenzhen Maternity and Child Health Care Hospital (Shenzhen Maternal and Child Ethics Review SFYLS [2022]094). All ethical regulations relevant to human research participants were followed. All procedures involving human participants were conducted in accordance with institutional guidelines and the Declaration of Helsinki.

### Sample collection and classification

#### Placental samples

Placentas were collected from eight singleton pregnancies and categorized into four groups: preterm controls, term controls, EOPE, and LOPE, with two cases in each group. The preterm control placentas were obtained from non-inflammatory preterm deliveries matched for gestational age with EOPE cases, and both mothers and infants were free of obstetric or medical complications. Term controls were collected from healthy women without obstetric or medical complications, matched for gestational age with LOPE cases. Diagnosis of preeclampsia followed the 2020 clinical guidelines issued by the Chinese Society of Obstetrics and Gynecology [7]. Full-thickness placental samples, including maternal decidua, chorionic villi, and fetal membranes, were harvested within 15 min after delivery.

#### Maternal serum samples

Peripheral blood was collected during early pregnancy (10–13 gestational weeks) from a prospective, independent cohort of 199 women, including 70 healthy controls, 32 EOPE cases, and 97 LOPE cases. Serum was separated and stored at −80  °C for untargeted metabolomic profiling. There was no overlap between the serum and placental cohorts. Clinical characteristics were obtained from the electronic files of the patients.

### Single-cell RNA sequencing and analysis

#### Tissue dissociation

Fresh placental tissue was washed twice with pre-chilled RPMI 1640 medium containing 0.04% BSA. Tissue was then finely chopped into approximately 0.5 mm³ pieces using surgical scissors and transferred to enzymatic digestion solution, which contained RPMI 1640, 0.04% BSA, and 0.2% collagenase II (Gibco, cat. no. 17101015). The tissue was incubated in a 37 °C incubator for 30–60 min with gentle mixing every 5–10 min. The digested suspension was filtered through a BD 40μm cell strainer (Falcon, cat. no. 352340) 1–2 times and centrifuged at 300×g for 5 min at 4  °C. The pellet was resuspended in an appropriate medium, mixed with an equal volume of red blood cell lysis buffer (Miltenyi, cat. no. 130-094-183), and incubated at 4  °C for 10 min. After centrifugation, the pellet was washed once with medium and the supernatant discarded. The cell concentration and viability were evaluated using a Luna-FL cell counter (Logos Biosystems, Korea).

#### Single-cell suspension preparation

The freshly prepared single-cell suspension was adjusted to a concentration of 700-1200 cells/μl. Library preparation and loading were performed following the manufacturer’s protocol for the 10× Genomics Chromium Next GEM Single Cell 3ʹ Reagent Kits v3.1 (cat.no. PN-1000268). Sequencing was performed on the Illumina Nova 6000 PE150 platform for high-throughput sequencing [8, 9].

#### Data acquisition and processing

Sequencing data were processed using the Cell Ranger (version 9.0.0) pipeline from 10x Genomics and aligned to the GRCh38 human reference genome to generate gene expression matrices.

#### Data quality control

Low-quality cells were filtered out using the Seurat (version 4.0.0) R package, with the following criteria: 1) Genes per cell > 200; 2) UMI count per cell > 1000; 3) log10GenesPerUMI > 0.7; 4) Mitochondrial gene proportion < 5%; 5) Hemoglobin gene proportion < 5%; 6) DoubletFinder (version 2.0.3) was used to remove potential doublets.

#### Data normalization and dimensionality reduction

Data were normalized using Seurat’s NormalizeData function with the “LogNormalize” method. The top 2000 highly variable genes (HVGs) were identified using Seurat’s FindVariableGenes function. PCA (Principal Component Analysis) was performed using RunPCA. Batch effects were corrected using the RunHarmony function in the Harmony (version 1.0) R package. UMAP (Uniform Manifold Approximation and Projection) was used for visualization.

#### Cell type identification and differential gene analysis

Marker genes for each cluster were identified using Seurat’s FindAllMarkers function. Differentially expressed genes (DEGs) were selected using the FindMarkers function with a threshold of |log₂FC | > 1, adjusted *P* < 0.05. GO (Gene Ontology) and KEGG (Kyoto Encyclopedia of Genes and Genomes) enrichment analyses were performed using R (version 4.0.3) based on the hypergeometric distribution.

### Pseudotime trajectory analysis

Cell developmental trajectories were reconstructed using Monocle. Cells were ordered in pseudotime space, and branches were assigned to early, intermediate, and late developmental states. Subcluster-specific pseudotime distributions were visualized, and stage-specific gene expression patterns were analyzed.

### Cell-cell communication analysis

Cell-cell communication networks were inferred using CellChat. Ligand-receptor interaction strength was calculated among all annotated cell types. Group-specific differences in signaling pathways—including IGF, CALCR, IL-10, and Chemerin—were visualized using circle plots and heatmaps. Communication strength shifts in preeclamptic versus control placentas were evaluated.

### Spatial transcriptomics and integration

#### Sample preparation for spatial transcriptomics

The placental tissue was stored at −80  °C prior to sectioning. Tissue was cut into 10 μm thick slices, which were mounted on 10x Genomics Visium CytAssist slides for spatial transcriptomics. The tissue sections were sequentially stained, fixed, and subjected to enzymatic digestion.

#### Library preparation and sequencing

The prepared tissue samples on the Visium CytAssist slide were subjected to RNA capture using oligo-dT primers that enabled the capture of poly-adenylated mRNA. The RNA was reverse-transcribed, followed by cDNA amplification. The resulting cDNA libraries were prepared for sequencing using Illumina sequencing technology [10, 11].

#### Data analysis and spatial deconvolution

The sequencing data generated from spatial transcriptomics were processed using the Space Ranger (version 2.1.0) software. This generated gene expression matrices for each spot on the tissue slide. The data were analyzed for spatially resolved gene expression patterns and regions of interest (ROIs) were identified by matching tissue morphology and gene expression profiles. Marker genes for spatial clusters were identified using FindMarkers (test.use = “presto”, |log₂FC | > 0.58, adjusted *P* < 0.05). Localized GO and KEGG enrichment analyses were conducted in spatially defined cell-dense regions, including Hofbauer cells, extravillous trophoblasts, and fibroblasts.

### Spatial metabolomics

#### Sample preparation for MSI

Placental tissue was stored at −80  °C and sectioned into 10 μm consecutive sagittal slices using a cryostat microtome (Leica CM 1950, Leica Microsystems, Germany). The tissue sections were thaw-mounted on positive charge desorption plates (Thermo Scientific, U.S.A) and stored at −80  °C until further analysis. The sections were desiccated at −20  °C for 1 h and at room temperature for 2 h before mass spectrometry imaging (MSI) analysis. An adjacent slice was used for hematoxylin-eosin (H&E) staining.

#### Mass spectrometry imaging (MSI) analysis

MSI analysis was performed using the AFADESI-MSI platform (Beijing Victor Technology Co., LTD, Beijing, China) coupled with a Q-Orbitrap Mass Spectrometer (Q Exactive, Thermo Scientific, U.S.A.). The solvent formulation was: Negative mode: acetonitrile (ACN) / H_2_O (8:2); Positive mode: acetonitrile (ACN) / H_2_O (8:2, 0.1% Formic Acid); Parameters for MSI: Solvent Flow Rate: 5 μL/min; Spray Voltage: 7 kV; Spray-to-Sample Distance: 3 mm; MS Resolution: 70,000; Mass Range: 70–1000 Da; AGC Target: 2E6; Capillary Temperature: 350  °C; The MSI analysis was conducted with a scanning rate of 0.2 mm/s in the x-direction and a 100 μm vertical step in the y-direction.

#### Data processing

Raw MSI data was processed using imzMLConverter to convert it into .imML format. The data were imported into MSiReader for ion image reconstruction, with background subtraction performed using the Cardinal 3 software. All MS images were normalized using Total Ion Count (TIC) in each pixel [12, 13]. High-spatial resolution H&E images were used to match regions of interest. Discriminating endogenous metabolites were identified using Orthogonal Partial Least Squares Discriminant Analysis (OPLS-DA), and VIP values were calculated for group discrimination. T-tests were performed for metabolite comparison between groups, with VIP > 1.0 and *p*-value < 0.05 as significant thresholds.

#### Metabolite identification

Metabolites detected during the AFADESI MSI analysis were annotated using the pySM pipeline and an in-house SmetDB database (Lumingbio, Shanghai, China).

### Serum untargeted metabolomics

#### Sample preparation and derivatization

Serum (150 μL) was mixed with 600 μL cold methanol-acetonitrile (2:1, v/v) containing internal standards (4 μg/mL), followed by vortexing, sonication, and protein precipitation at −40  °C. Supernatants were split for LC-MS and GC-MS. For GC-MS, samples were derivatized with methoxyamine hydrochloride and BSTFA.

#### Instrumentation and analysis

LC-MS was performed on an ACQUITY UPLC HSS T3 column with water (0.1% formic acid) and acetonitrile at 0.35 mL/min. GC-MS used a DB-5MS column with helium carrier gas and a programmed temperature gradient. MS/MS was conducted on an AB Sciex TripleTOF 6600 in both ESI ionization modes.

#### Data processing

Raw LC-MS data were converted to mzML format using ProteoWizard. XCMS was used for peak extraction, retention time correction, and alignment. Signal correction was performed using support vector regression (SVR) [14]. Peaks with >50% missing data were excluded. Metabolite annotation was conducted using in-house libraries, HMDB, Metlin, and MetDNA.

### Predictive modeling of preeclampsia using serum metabolites

Serum samples were analyzed to identify potential biomarkers for preeclampsia using logistic regression modeling. Univariate logistic regression was first performed to identify metabolites significantly associated with preeclampsia (*p* < 0.05). Variables with *p*-values < 0.05 were then included in a multivariable logistic regression model. The dataset was split into training (70%) and validation (30%) sets, with a random seed (1234) applied for reproducibility. Stepwise bidirectional regression analysis was conducted to identify significant predictors of early-onset preeclampsia. Internal validation of the model was performed by evaluating its performance on the validation set, with Receiver Operating Characteristic (ROC) curves and Area Under the Curve (AUC) values calculated for both sets. Additional evaluations included a nomogram, calibration curves, decision curve analysis (DCA), and confusion matrices to assess the model’s clinical utility and diagnostic performance.

### Statistical analysis

Statistical analysis was performed in R (v4.0.3). Cell proportions and pathway activity scores were compared using Student’s t-test or Wilcoxon rank-sum test, with Bonferroni correction for multiple testing. Group-wise clinical comparisons were conducted using one-way ANOVA or Kruskal-Wallis tests.

## Results

### Single-cell transcriptomic atlas of preeclamptic and control placentas

**Fig. 1:**
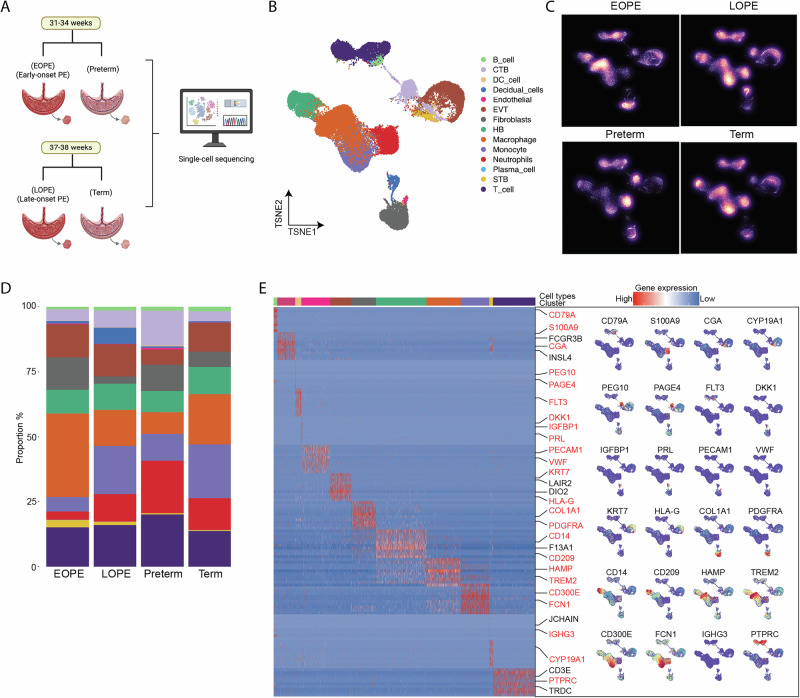
Single-cell landscape of the placentas in control and preeclampsia. **A** Schematic illustration of the workflow. Single-cell transcriptome (scRNA-seq) analysis was conducted in eight placentas from the pregnancy with control (preterm and term), and preeclampsia (early-onset and late-onset preeclampsia). **B** Cell type identification based on scRNA-seq data. A total of 14 cell types were identified. **C** The density plot of scRNA-seq data in 4 groups of placentas. **D** The proportion of identified cell types in the placentas. **E** Heatmap showing the scaled expression levels of cell type-specific marker genes. The expression patterns of 24 representative marker genes are shown on the right. EOPE, early-onset preeclampsia; LOPE, late-onset preeclampsia; DC cell, dendritic cells; HB, Hofbauer Cell; CTB, cytotrophoblast; EVT, extravillous trophoblast; STB, syncytiotrophoblast

To generate a reference cell map of preeclamptic placentas, we performed single-cell RNA sequencing (scRNA-seq) using the 10x Genomics platform, analyzing a total of 75,092 cells derived from eight placentas, including four from control pregnancies (two preterm, two term) and four from preeclamptic pregnancies (two early-onset, two late-onset). A simplified workflow is presented in Fig. 1A. All samples were obtained from Chinese mothers, with no significant differences in clinical characteristics between the control and preeclamptic groups (Supplementary Table 1). The mean maternal age was 30 years, with an average gestational age at delivery of 35 weeks, and the majority of participants were primiparous. Although birth weight was lower in the preeclampsia group compared to controls, this difference did not reach statistical significance.

We identified the primary cell types within placental tissues using t-distributed stochastic neighbor embedding (t-SNE) analysis. These included B cells, cytotrophoblasts (CTB), dendritic cells (DC), decidual cells, endothelial cells, extravillous trophoblasts (EVT), fibroblasts, Hofbauer cells, macrophages, monocytes, neutrophils, plasma cells, syncytiotrophoblasts (STB), and T cells (Fig. 1B). A density plot revealed that immune cells, such as HB, macrophages, neutrophils, and T cells, along with CTB, exhibited the most robust signals (Fig. 1C). Notably, macrophages constituted over 30% of the total cell population in EOPE group (early-onset preeclamptic placentas), a proportion that was reduced to less than 10% in preterm group (preterm placentas, Fig. 1D). Furthermore, the EOPE group demonstrated reduced proportions of CTB, EVT, monocytes, neutrophils, and T cells, along with an increased proportion of STB compared to the preterm group (Fig. 1D). By contrast, the LOPE group (late-onset preeclamptic placentas) exhibited a cellular distribution largely comparable to that of the term group (term placentas), with the exception of higher levels of CTB and decidual cells (Fig. 1D). These cells were further classified into 14 transcriptionally distinct clusters, each defined by unique cluster-specific markers (Fig. 1E).

### Hofbauer cell subclusters exhibit immune and developmental alterations in preeclampsia

**Fig. 2:**
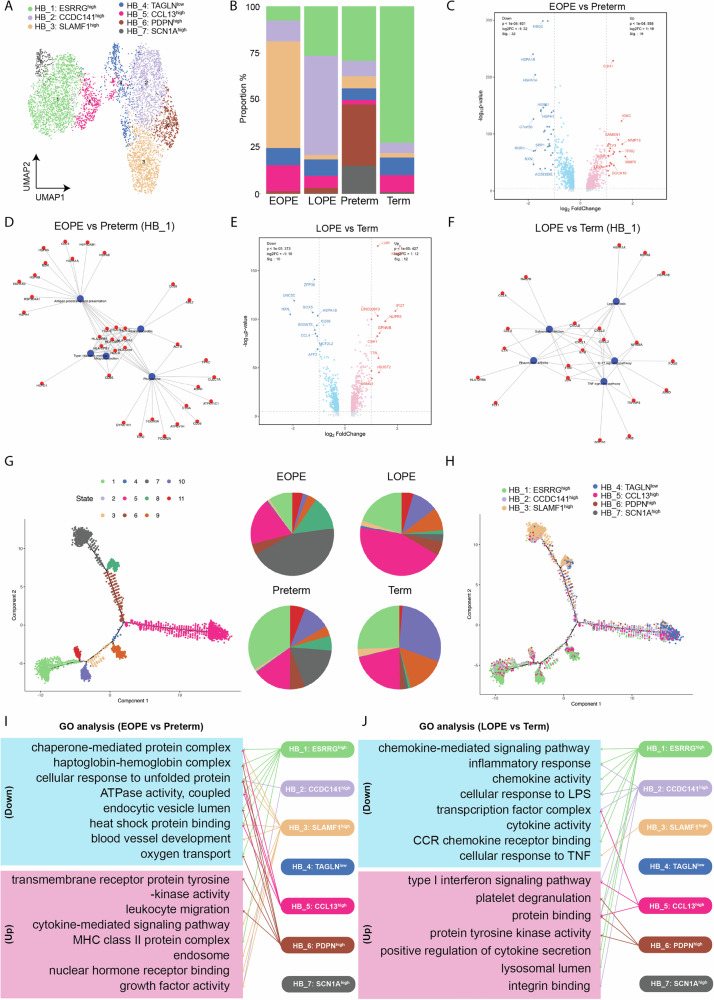
Heterogeneity of Hofbauer cells in control and preeclampsia placentas. **A** Identification of Hofbauer cell types based on scRNA-seq data revealed a total of seven distinct cell types. **B** Proportions of the identified HB cell types in the placentas were analyzed. **C** Volcano plot illustrating differentially expressed genes (DEGs) in Hofbauer cells between the EOPE and preterm groups. **D** Cnetplot depicting the top five Gene Ontology (GO) terms associated with upregulated genes in the preterm group. **E** Volcano plot of DEGs in Hofbauer cells between the LOPE and term groups. **F** Cnetplot showing the top five GO terms for upregulated genes in the term group. **G** Branch trajectories were divided into 11 cellular states, with comparisons of cell proportions across the four groups, with color coding to denote the cell states. **H** Inference of pseudotime trajectories in Hofbauer cells, color-coded according to the corresponding cell subsets. **I** GO analysis of each HB cluster between the EOPE and preterm groups. **J** GO analysis of each HB cluster between the LOPE and term groups. EOPE early-onset preeclampsia, LOPE late-onset preeclampsia, HB Hofbauer Cell

Unsupervised clustering of Hofbauer cells revealed seven transcriptionally distinct subclusters (HB_1 to HB_7; Fig. 2A). In the preterm group, HB_1, HB_6, and HB_7 predominated, but their proportions declined markedly in EOPE group, which showed expansion of HB_2 and HB_5 (Fig. 2B). Similarly, in term placentas, HB_1 dominated in term group but was replaced by HB_2 in LOPE group, accounting for over 50% of Hofbauer cells.

DEGs analysis revealed that HB_1 in EOPE group was enriched in antigen presentation and phagosome pathways, while HB_1 in LOPE group was associated with TNF, IL-17, and rheumatoid arthritis signaling (Fig. 2C–F). Additional subclusters displayed enrichment in immune activation, ferroptosis, and infection-related pathways (Supplementary Fig. 1A–E). Trajectory analysis using Monocle revealed shifts in developmental dynamics: >50% of EOPE Hofbauer cells remained in early pseudotime, whereas most cells of preterm group were in the late state (Fig. 2G–H). Subclusters HB_3 and HB_6 were enriched in early trajectories, while HB_2, HB_4, and HB_5 predominated at intermediate stages. Gene Ontology analysis highlighted unique functional programs: in EOPE group, HB_1 and HB_6 were associated with oxygen transport and hemoglobin-related processes, while HB_2 showed unfolded protein response enrichment (Fig. 2I). In LOPE group, HB_1 and HB_3 were involved in inflammatory cytokine responses, while HB_5 and HB_6 showed enrichment in interferon and platelet degranulation signaling (Fig. 2J).

### Trophoblast subtypes reveal dysregulated differentiation and hypoxic signatures

**Fig. 3:**
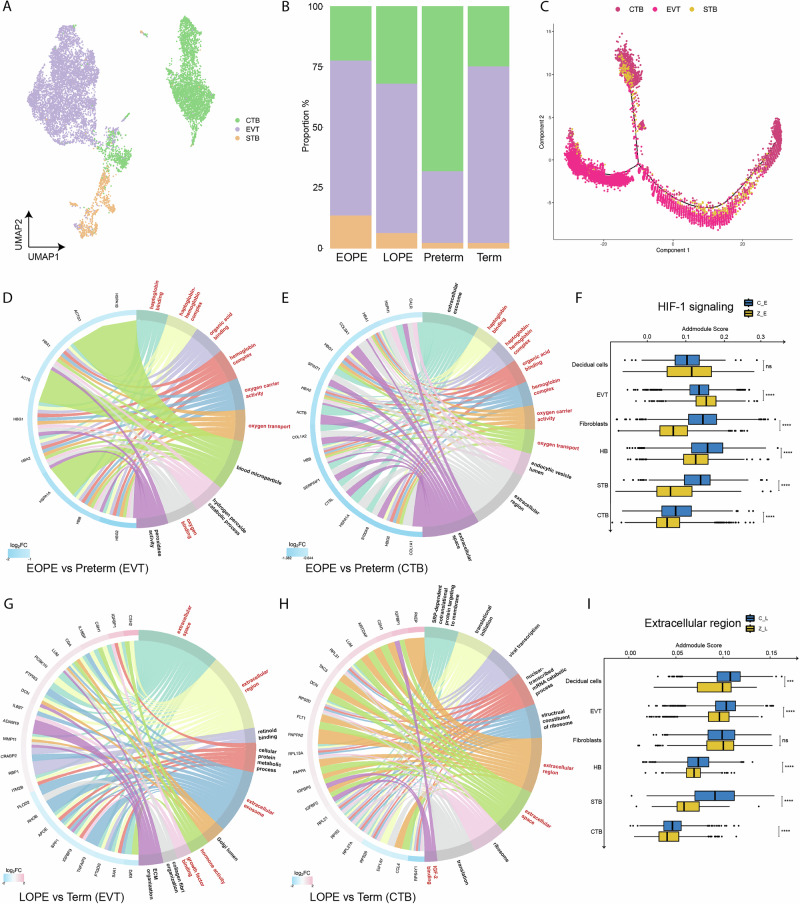
Heterogeneity of trophoblast cells in control and preeclampsia. **A** A total of three major trophoblast cell types were identified. **B** The proportion of identified trophoblast cell types in the placentas. **C** Inference of pseudotime trajectories for trophoblast cells, color-coded according to cell subsets. **D** Gene Ontology (GO) analysis of extravillous trophoblast (EVT) cells between EOPE and preterm group. **E** GO analysis of cytotrophoblast (CTB) cells between EOPE and preterm group. **F** The expression of HIF-1 in various placental cells between EOPE and preterm group. **G** GO analysis of EVT cells between LOPE and term group. **H** GO analysis of CTB cells between LOPE and term group. **I** The expression of gene related to extracellular region in various placental cells between EOPE and preterm group. EOPE early-onset preeclampsia, LOPE late-onset preeclampsia, CTB cytotrophoblast, EVT extravillous trophoblast, STB syncytiotrophoblast, HB Hofbauer cells. ***, *P* < 0.001; ****, *P* < 0.0001; ns not significant

Trophoblasts were classified into CTB, EVT, and STB clusters (Fig. 3A). EVT was the dominant subtype in most samples except preterm group, which had a larger CTB population (Fig. 3B). The EOPE group showed a reduction in CTB with compensatory increases in EVT and STB. In contrast, LOPE group showed elevated CTB but reduced EVT compared to term group, suggesting impaired trophoblast differentiation. Monocle analysis revealed that EVT resided in late pseudotime, while CTB and STB appeared in earlier states (Fig. 3C), consistent with their developmental hierarchy.

In early-onset preeclampsia, DEGs in EVT and CTB were enriched in pathways related to hemoglobin binding, oxygen transport, and oxidative stress (Fig. 3D, E). Notably, HIF-1 signaling showed cell-type-specific dysregulation, with decreased expression in decidual and EVT cells, but increased expression in CTB, STB, and HB cells in the EOPE group (Fig. 3F).

In late-onset preeclampsia, trophoblasts exhibited enrichment in extracellular region, IGF binding, hormone activity, and ECM remodeling pathways (Fig. 3G, H). A gene module analysis revealed a global upregulation of extracellular region-related genes across all cell types in LOPE group, including CTB, EVT, and STB (Fig. 3I), suggesting broad matrix remodeling and altered growth factor bioavailability.

### Localized cell-cell interactions reveal subtype-specific microenvironmental remodeling

**Fig. 4:**
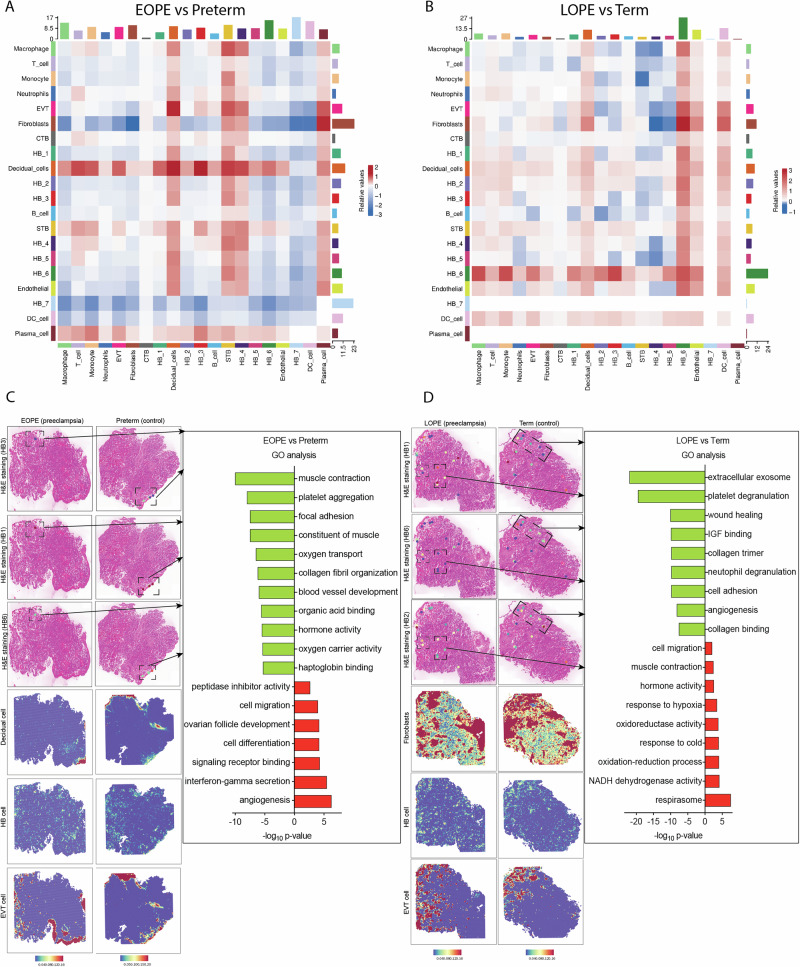
Cell interaction between Hofbauer cell subclusters and other cells. **A** Differential interaction strength heatmap in EOPE and preterm group by Cellchat analysis. **B** Differential interaction strength heatmap in LOPE and term group by Cellchat analysis. **C** Local GO analysis of the interface with HB subclusters and EVT accumulation in EOPE and preterm group by spatial transcriptomics analysis. **D** Local GO analysis of the interface with HB subclusters and EVT accumulation in LOPE and term group by spatial transcriptomics analysis. EOPE early-onset preeclampsia, LOPE late-onset preeclampsia, HB Hofbauer cell, EVT extravillous trophoblast

To investigate altered cell-cell interactions, we constructed a ligand-receptor communication network across major placental cell types. In EOPE group, strong interactions were observed between decidual cells and specific Hofbauer cell subtypes (HB_1, HB_3, HB_6), as well as with EVT and STB (Fig. 4A). In contrast, LOPE group showed prominent interactions between HB_6 and EVT, fibroblasts, and macrophages (Fig. 4B), indicating distinct microenvironmental adaptations.

Spatial transcriptomics further confirmed these findings. Cell types were deconvoluted using Robust Cell Type Decomposition (RCTD), and DEGs were mapped in spatially enriched regions. In EOPE group, spatial zones with high densities of HB_1, HB_3, HB_6, EVT, and decidual cells exhibited downregulation of muscle contraction, oxygen transport, platelet aggregation, and blood vessel development, alongside upregulation of angiogenesis, interferon-γ signaling, and cell migration (Fig. 4C). In LOPE group, regions with abundant HB_1, HB_2, HB_6, EVT, and fibroblasts showed suppression of extracellular vesicle secretion, platelet degranulation, wound healing, and IGF binding. Upregulated pathways included hypoxia response, oxidative phosphorylation (NADH dehydrogenase activity), and mitochondrial metabolism (Fig. 4D). These findings highlight differential remodeling of the placental microenvironment across preeclampsia subtypes.

### Metabolic reprogramming in Hofbauer cells and trophoblasts

**Fig. 5:**
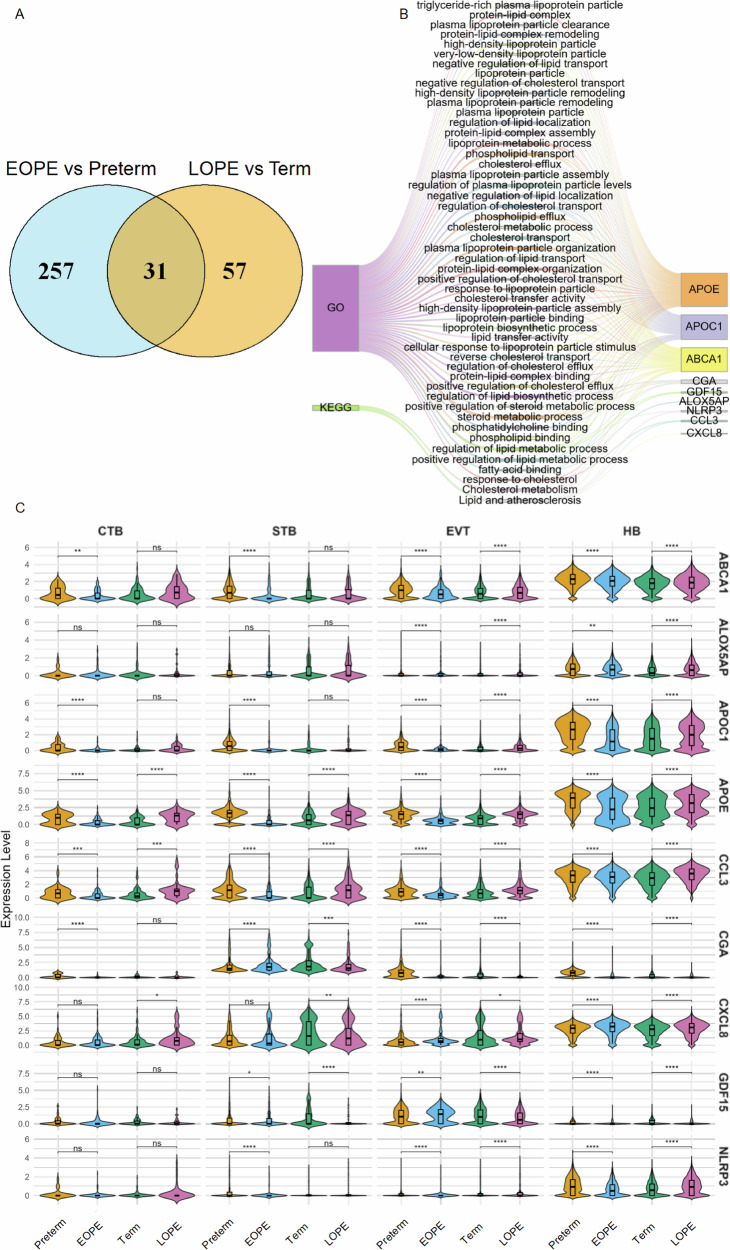
Single-cell transcriptomic analysis reveals cell-type-specific metabolic gene dysregulation in preeclamptic placentas. **A** Venn diagram depicting the overlap of DEGs between EOPE vs. preterm (blue circle) and LOPE vs. term (orange circle), as identified by scRNA-seq. The overlapping region (brown) represents shared DEGs dysregulated in both subtypes of preeclampsia. **B** GO and KEGG enrichment analysis of the 31 overlapping DEGs. The Sankey diagram only illustrates enriched lipid metabolism-related pathways with key regulators. **C** Violin plots displaying the expression profiles of selected lipid metabolism and inflammation-related genes across CTB, STB, EVT, HB cells derived from scRNA-seq data. Gene expression levels are compared between disease and gestational age-matched control groups: EOPE (orange) versus preterm (blue), and LOPE (green) versus term (purple). Statistical comparisons were conducted within each cell type between the respective disease and control groups. ns, not significant; *, *P* < 0.05; **, *P* < 0.01; ***, *P* < 0.001; ***, *P* < 0.0001. EOPE early-onset preeclampsia, LOPE late-onset preeclampsia

Given the altered cellular interactions and functional states identified in Hofbauer cells and trophoblasts, we hypothesized that local metabolic disturbances may contribute to the disrupted microenvironment observed in preeclampsia. To test this hypothesis, we leveraged our single-cell transcriptomic dataset to profile metabolic gene expression across four clinical groups. Differential expression analysis revealed 31 genes consistently dysregulated in both preterm vs. EOPE and term vs. LOPE comparisons (Fig. 5A). GO and KEGG enrichment analyses showed that these shared DEGs were predominantly involved in lipid metabolism-related pathways, including cholesterol efflux, lipoprotein particle remodeling, and clearance of triglyceride-rich lipoproteins (Fig. 5B), suggesting that impaired lipid processing may be a key metabolic hallmark of the preeclamptic placenta. Among the enriched genes, APOE, APOC1, and ABCA1 emerged as central regulators of lipid transport and homeostasis.

We then examined the expression of these genes across CTB, STB, EVT, and HB cells using our scRNA-seq data (Fig. 5C). Among them, EVT and HB cells showed the most prominent and consistent changes, highlighting their central role in the pathophysiology of preeclampsia. Notably, within EVT cells, several lipid metabolism genes such as ABCA1, APOC1, and APOE were upregulated in EOPE group but showed downregulation in LOPE group, suggesting a stage-specific shift in metabolic function. In contrast, GDF15, which was downregulated in early-onset EVT, displayed increased expression in late-onset cases, indicating a reversal in stress response signaling. In HB cells, the expression patterns were relatively more consistent across disease subtypes, but not without exceptions. For instance, APOC1 and APOE exhibited opposite regulation trends between early-and late-onset groups, reinforcing the notion of subtype-specific immune-metabolic remodeling. Collectively, these findings indicate that both early-and late-onset preeclampsia are associated with dysregulation of metabolic and inflammatory pathways, yet the direction and magnitude of these changes differ by cell type and disease subtype, underscoring the molecular heterogeneity underlying preeclampsia.

### Integrated spatial and circulating metabolomics reveal metabolic dysregulation and predictive signatures in preeclampsia

To validate whether the transcriptional alterations observed in Hofbauer cells and trophoblasts lead to functional metabolic perturbations at the tissue and systemic levels, we conducted integrated metabolomic analyses. Specifically, we applied a two-pronged approach combining spatial metabolomics of placental tissue with serum metabolomic profiling from an independent cohort to assess metabolic disruptions and their potential diagnostic value in preeclampsia.

**Fig. 6:**
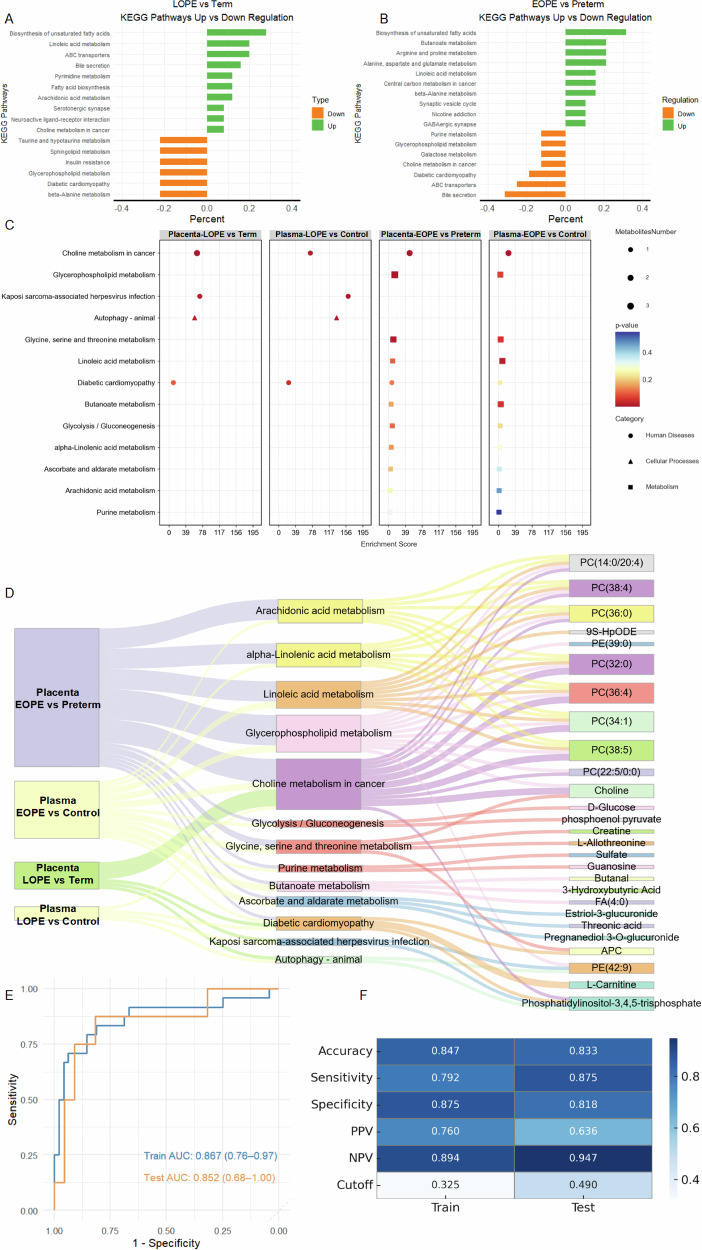
Spatial and circulating metabolite signatures associated with preeclampsia. **A**, **B** KEGG pathway enrichment analysis comparing late-onset (LOPE vs. term, **A** and early-onset (EOPE vs. preterm, **B** preeclampsia placentas. Upregulated (orange) and downregulated (green) pathways are shown based on fold-change direction and enrichment percent. **C** Shared enriched metabolic pathways in placenta and serum across early- and late-onset preeclampsia groups. Dot size represents the number of metabolites involved; color indicates pathway category. **D** Sankey plot illustrating the correspondence between key enriched pathways and specific metabolites across placental and serum compartments in both early-onset and late-onset preeclampsia. **E** ROC curves of the three-metabolite diagnostic model in the training and validation cohorts. The blue curve represents the training cohort, while the orange curve represents the validation cohort. **F** Heatmap summarizing performance metrics, including accuracy, sensitivity, specificity, positive predictive value (PPV), negative predictive value (NPV), and optimal cutoff of the diagnostic model in the training and validation cohorts. EOPE, early-onset preeclampsia; LOPE, late-onset preeclampsia

Spatial metabolomics performed on frozen placental sections revealed region-specific metabolic alterations, with EOPE placentas showing elevated levels of positively charged metabolites and reduced negatively charged metabolites compared to preterm controls (Supplementary Fig. 2A), indicating a disturbed local metabolic environment. In contrast, no substantial differences were observed between term and LOPE placentas (Supplementary Fig. 2B), suggesting that metabolic disruption is more pronounced in the early-onset form of the disease. Subsequent pathway enrichment analysis revealed that EOPE placentas showed upregulation of biosynthesis of unsaturated fatty acids, arginine and proline metabolism, and butanoate metabolism, along with downregulation of bile secretion and ATP-binding cassette (ABC) transporter pathways (Fig. 6A). In the LOPE group, enrichment was observed in linoleic acid metabolism, bile secretion, and ABC transporter activity, whereas pathways related to insulin resistance and sphingolipid metabolism were suppressed (Fig. 6B). These spatial metabolic signatures were concordant with transcriptomic findings, reinforcing the role of lipid and energy metabolism dysregulation in the placental pathology of preeclampsia.

To evaluate whether such placental metabolic disturbances are reflected systemically, we conducted untargeted serum metabolomics on an independent cohort comprising 199 early-pregnancy serum samples (10–13 gestational weeks), including women who later developed early-onset or late-onset preeclampsia and matched controls (Supplementary Table 2). Volcano plot analyses identified significantly dysregulated metabolites in both EOPE and LOPE groups compared to controls (Supplementary Fig. 3A&3B). KEGG pathway enrichment of these serum metabolites, when intersected with spatial metabolomics results, revealed several shared metabolic pathways between the placenta and maternal serum, particularly in EOPE (Fig. 6C). Sankey diagrams were subsequently constructed to illustrate the flow and overlap of enriched metabolic pathways and corresponding metabolites across placental and circulating compartments in both early- and late-onset preeclampsia (Fig. 6D). These visualizations emphasized not only the shared pathways but also the disease-subtype specific features of metabolic dysregulation, reinforcing the systemic relevance of placental metabolic shifts.

Given the goal of identifying convenient circulating biomarkers for disease prediction, we prioritized serum metabolites. As only one intersecting metabolite was found for LOPE, modeling efforts focused on the 11 differential serum metabolites associated with EOPE. Boxplots (Supplementary Fig. 3C–M) demonstrated significant differences in the levels of these metabolites between EOPE and control. Subsequently, univariate and multivariate logistic regression analyses were conducted. After stepwise selection, three metabolites, PC(22:5/0:0), 3-Hydroxybutyric Acid, and L-Allothreonine were retained for final model construction (Supplementary Table 3).

The dataset was randomly split into a training set (70%) and a validation set (30%). The resulting model showed good discriminative performance, achieving an AUC of 0.867 (95% CI: 0.76-0.97) in the training set and 0.852 (95% CI: 0.68–1.00) in the validation set (Fig. 6E). To facilitate individualized risk prediction, a nomogram based on the three selected metabolites was developed (Supplementary Fig. 3N). Calibration curves (Supplementary Fig. 3O, P) further confirmed good agreement between predicted and observed outcomes, with Hosmer-Lemeshow *p*-values of 1.000 and 0.008 for the training and validation cohorts, respectively. Clinical utility was assessed through Decision Curve Analysis (DCA), which demonstrated favorable net benefits across a range of threshold probabilities in both training (Supplementary Fig. 3Q) and validation (Supplementary Fig. 3R) sets. A heatmap was generated to visualize model performance across both sets, including accuracy, sensitivity, specificity, positive predictive value (PPV), and negative predictive value (NPV), further highlighting the balanced and robust predictive capacity of the model (Fig. 6F).

## Discussion

Preeclampsia is traditionally divided into early- and late-onset subtypes based on gestational age [1], but these differ not only in timing, but also in placental pathology, severity, and clinical outcomes. In this study, we integrated single-cell, spatial transcriptomic, and spatial metabolomic analyses of placental tissues with early-pregnancy serum metabolomics to dissect cellular and metabolic heterogeneity across subtypes. Although the placental and serum analyses were performed at different gestational stages, they were designed to be complementary: the early-pregnancy serum profiles captured systemic metabolic alterations preceding disease onset, while the placental multi-omics reflected endpoint tissue changes. The convergence of lipid-transport and mitochondrial-stress pathways across these layers provides hypothesis-generating evidence linking early metabolic stress to later placental remodeling rather than indicating direct temporal causality. The analysis highlighted trophoblasts and Hofbauer cells as key sites of immune-metabolic disturbance and revealed lipid-transport and mitochondrial-stress pathways as potential molecular bridges between placental dysfunction and maternal metabolism.

Although early- and late-onset preeclampsia often share hypertension, proteinuria, and maternal organ dysfunction [1, 2, 4, 5], early-onset preeclampsia is associated with more severe placental damage and clinical outcomes [15–17]. Our data showed early-onset preeclampsia placentas had increased macrophages and EVTs and reduced neutrophils, suggesting an imbalance in immune cell populations [18, 19]. In contrast, late-onset preeclampsia exhibited a cellular composition closer to that of term controls, but with a more pro-inflammatory transcriptional profile enriched for TNF, NF-κB, and chemokine signaling [15, 20].

Hofbauer cells, the fetal macrophages of the placenta, are crucial for immune tolerance and vascular remodeling [21, 22]. We identified HB_1 (ESRRG^high) and HB_6 (PDPN^high) subtypes involved in oxygen transport and matrix interaction. These subpopulations were significantly reduced in early-onset preeclampsia placentas. ESRRG, a nuclear receptor regulating mitochondrial oxidative phosphorylation [23], and PDPN, involved in lymphangiogenesis and stromal interaction [24–26], likely mediate placental adaptation to hypoxia and endothelial remodeling. Their depletion may contribute to EVT invasion defects and spiral artery remodeling failure.

Trophoblast imbalance was also evident. early-onset preeclampsia placentas had fewer CTBs but more STBs and EVTs, suggesting premature differentiation. These cells exhibited reduced oxygen-transport capacity and upregulated HIF-1 signaling, VEGF, and IGF pathways, reflecting stress-induced placental adaptation [27–30]. In late-onset preeclampsia, CTBs accumulated while EVT proportions declined, alongside enrichment in extracellular matrix and IGF-binding pathways, indicating impaired differentiation and matrix-mediated invasion [30].

Spatial transcriptomics highlighted microenvironmental remodeling. In early-onset preeclampsia, placental regions with dense Hofbauer and EVT cell populations showed downregulation of oxygen transport and muscle contraction, with upregulation of angiogenesis, interferon-γ response, and cell migration. These changes imply compensatory but insufficient vascular development. Interferon-γ likely induces EVT apoptosis and Hofbauer cell activation, sustaining local inflammation [31–33]. In late-onset preeclampsia, disruptions in exosome secretion, IGF binding, and wound healing were accompanied by oxidative phosphorylation and NADH dehydrogenase activity, suggesting mitochondrial stress.

Transcriptional profiling of Hofbauer cells and trophoblasts further revealed subtype-specific metabolic reprogramming. Genes involved in lipid transport (e.g., APOE, APOC1, ABCA1), oxidative phosphorylation, and inflammation (e.g., NLRP3, GDF15) were differentially expressed. GDF15, previously reported to be elevated in the maternal circulation of preeclampsia [34], showed reduced placental expression in our dataset, suggesting that systemic metabolic stress is amplified as local adaptive capacity declines. Early-onset preeclampsia placentas showed stronger perturbations, indicating that immune-metabolic dysfunction underlies its pathogenesis. Spatial metabolomics confirmed metabolic reprogramming. Early-onset preeclampsia placentas demonstrated elevated levels of fatty acid oxidation intermediates and amino acid catabolites, and reduced tricarboxylic acid cycle and anti-inflammatory metabolites [16, 35–39]. Suppressed ABC transporter and arginine metabolism pathways further supported vascular dysfunction [40–43]. Late-onset preeclampsia placentas displayed altered bile and linoleic acid metabolism and increased oxidative stress markers [37, 44–46].

Notably, the divergent regulation of lipid-transport genes such as ABCA1, APOC1, and APOE between early- and late-onset preeclampsia indicates a stage-dependent metabolic adaptation in extravillous trophoblast (EVT) cells. In EOPE, their upregulation is consistent with enhanced cholesterol efflux and lipoprotein remodeling under hypoxic stress, reflecting a transient compensatory response that helps maintain trophoblast energy balance. ABCA1-mediated cholesterol export to APOE supports placental lipid homeostasis [47], whereas its impairment contributes to abnormal lipid accumulation and vascular stress in preeclampsia [48]. In contrast, the downregulation of these pathways in LOPE suggests loss of this adaptive capacity, possibly driven by sustained oxidative and inflammatory stress. This stage-dependent divergence supports the view that early placentas activate lipid transport as a protective mechanism against hypoxic injury, whereas late placentas experience metabolic exhaustion that results in mitochondrial dysfunction and impaired EVT invasion-a process recognized as central to preeclampsia pathophysiology [49].

Building upon these transcriptomic and metabolic observations, our results suggest that disrupted lipid utilization and mitochondrial stress jointly drive progressive placental maladaptation. In early stages, trophoblasts enhance lipid turnover to sustain ATP production under hypoxia, representing a transient adaptive response. However, prolonged oxidative stress depletes this capacity, leading to mitochondrial injury and excessive reactive oxygen species [50]. Damaged trophoblasts may release lipid mediators that activate nearby Hofbauer cells, forming a self-perpetuating inflammatory circuit within the villous core [51]. This trophoblast-macrophage crosstalk, previously shown to induce inflammatory polarization and impair invasion [52], supports a model in which metabolic compensation gradually evolves into chronic energy exhaustion and immune activation, ultimately leading to placental dysfunction in preeclampsia.

To bridge placental changes with systemic biomarkers, we performed serum metabolomics in early pregnancy. In early-onset preeclampsia, three metabolites (PC(22:5/0:0), 3-hydroxybutyric acid, and L-allothreonine) emerged as significant predictors. These reflect mitochondrial stress, ketone body metabolism, and ECM turnover [27, 45]. Logistic regression models based on these markers yielded high accuracy (AUC > 0.85). In contrast, late-onset preeclampsia showed minimal serum-placental overlap, limiting its early prediction.

This work provides encouraging, multi-layered insights yet remains an initial exploration of multi-omics characteristics and therefore has limitations. The placental cohort was small, and the serum and placental datasets were not paired, which restricts causal inference. Independent validation using external cohorts was not performed, and functional experiments to confirm mechanistic links are still needed. Therefore, future studies with larger, longitudinal, and mechanistic designs are warranted to substantiate these findings.

### Perspective of Asia

This multi-omics study in Chinese women demonstrates that EOPE and LOPE share core immune-metabolic disturbances while exhibiting distinct subtype-specific features, highlighting the biological heterogeneity of preeclampsia in East Asian populations. An early-pregnancy EOPE prediction model based on serum metabolite signatures was also developed, and further evaluation in broader Asian cohorts will be essential to confirm its translational applicability.

## Conclusion

In conclusion, this integrative multi-omics study delineates both shared and divergent molecular landscapes between early- and late-onset preeclampsia. Hofbauer cell heterogeneity and trophoblast differentiation emerged as pivotal processes shaping subtype-specific pathology. The observed immune-metabolic remodeling, characterized by lipid dysregulation and oxidative stress, indicates stage-dependent adaptive and maladaptive responses. Moreover, the identification of early-pregnancy serum metabolites associated with early-onset preeclampsia offers preliminary support for non-invasive early risk assessment. Together, these findings generate testable hypotheses and establish a framework for future mechanistic and translational research aimed at advancing precision prenatal care.

## Supplementary information

Supplementary information

## Data Availability

The data that support the findings of this study are available from the corresponding author upon reasonable request.
