# Supplementary material for: Subtype specific immune-metabolic reprogramming in preeclampsia revealed by multiomics and serum biomarkers
Source: Hypertens Res. 2025 Dec 19;49(3):641–57. doi: 10.1038/s41440-025-02504-5 (PMC12960252; doi:10.1038/s41440-025-02504-5)
Supplement: Supplementary file 1 — Supplementary information [file 41440_2025_2504_MOESM1_ESM.docx]

**Subtype Specific Immune-Metabolic Reprogramming in Preeclampsia Revealed by Multiomics and Serum Biomarkers**

Yixuan Chen^1*^. Linlin Wu^2*^. Dongni Huang^3*^. Xiaoxia Wu^4^. Kan Liu^5^. Bo Sun^6^. Jinying Yang^7^. Baozhen Zhang^3^. Zijun Ouyang^8^. Cuilian Zhang^1#^. Lunbo Tan^3#^. Jianmin Niu^4#^

1. Department of Reproductive Medical Center. Henan Provincial People's Hospital. People's Hospital of Zhengzhou University. Henan Provincial People's Hospital of Henan University. 7 Weiwu Road. Zhengzhou. Henan. 450003. China.

2. Department of Obstetrics. The Eight Affiliated Hospital. Sun Yat-Sen University. 3025 Shennan Middle Road. Shenzhen 518033. China.

3. Women and Children's Hospital of Chongqing Medical University. Chongqing 401147. China.

4. Department of Obstetrics. Shenzhen Maternity & Child Healthcare Hospital. 2004 Hongli Road. Shenzhen. Guangdong. 518028. China.

5. Department of Obstetrics. Henan Provincial People's Hospital. People's Hospital of Zhengzhou University. Henan Provincial People's Hospital of Henan University. 7 Weiwu Road. Zhengzhou. Henan. China.

6. Department of Obstetrics. Shenzhen Baoan Women's and Children's Hospital. Shenzhen. 518100. China.

7. Department of Obstetrics. Longgang Maternity and Child Clinical Institute. Shenzhen. 518172. China.

8. School of Food and Drug. Shenzhen Polytechnic University. 7098 Liuxian Avenue. Shenzhen 518055. China.

* Yixuan Chen. Linlin Wu and Dongni Huang contributed equally to this work.

# Corresponding authors: Jianmin Niu (E-mail: njianmin@163.com). Lunbo Tan (E-mail: lunbo.tan@outlook.com) and Cuilian Zhang (E-mail: luckyzcl@qq.com).

**Supplementary Table 1**. Characteristics of the patients whose placentas were used for multi-omics analysis.

| Factors | Control | Preeclampsia |
| --- | --- | --- |
|  |  |  |
| N | 4 | 4 |
| Maternal age (years) | 30 (21-37) | 30 (23-37) |
| Asian. n | 4 | 4 |
| Pre-term or early-onset. n | 2 | 2 |
| Fetal sex (n female/male) | 3/1 | 3/1 |
| Gestational age at delivery (weeks) | 35.7 (33.0-38.1) | 35.0 (31.5-38.1) |
| Parity. n | 1 (1-2) | 1 (1-1) |
| Birth weight (g) | 2440 (1605-3025) | 1958 (1460-2443) |

Data are presented as mean (interquartile range) or number.

**Supplementary Table 2**. Characteristics of the patients whose serum were used for non-targeted metabolomics analysis.

| Factors | Control | Early-onset Preeclampsia | Late-onset Preeclampsia |
| --- | --- | --- | --- |
| N | 70 | 32 | 97 |
| Maternal age (years) | 32 (29-36) | 33 (29-37) | 32 (28-36) |
| Gestational age at sampling (weeks) | 10.6 (8.8-12.4) | 12.0 (10.7-14.7) | 12.4 (9.0-12.4) |
| Gestational age at delivery (weeks) | 39.5 (38.8-40.4) | 33.2** (32.4-34.0) | 37.8** (36.6-39.1) |

Data are presented as mean (interquartile range) or number. One-way ANOVA or Kruskal-Wallis test is used for comparing the significant difference among three group. **. P<0.01.

**Supplementary Table 3.** Univariate and multivariate logistic regression analysis of early-onset preeclampsia-related serum metabolites.

| Variables | Univariate | |  | Multivariate | |
| --- | --- | --- | --- | --- | --- |
|  | *P* | OR (95%CI) |  | *P* | OR (95%CI) |
| PC(14:0/20:4) | 0.8681 (0.71–1.06) | 0.1657 |  |  |  |
| PC(22:5/0:0) | 3.8400 (1.63–9.07) | 0.0021 |  | 3.2136 (1.1400–9.0400) | 0.0269 |
| 9S-HpODE | 0.7904 (0.53–1.18) | 0.2522 |  |  |  |
| 3-Hydroxybutyric Acid | 0.4588 (0.26–0.80) | 0.0058 |  | 0.3627 (0.1800–0.7500) | 0.0061 |
| Butanal | 0.5267 (0.32–0.85) | 0.0094 |  |  |  |
| APC | 0.6004 (0.40–0.91) | 0.0165 |  |  |  |
| L-Allothreonine | 4.5690 (1.75–11.92) | 0.0019 |  | 4.5314 (1.4500–14.1800) | 0.0094 |
| phosphoenol pyruvate | 1.2263 (0.89–1.69) | 0.2123 |  |  |  |
| Pregnanediol 3-O-glucuronide | 1.7757 (1.07–2.94) | 0.0258 |  |  |  |
| Estriol-3-glucuronide | 1.2912 (0.96–1.73) | 0.0858 |  |  |  |
| Sulfate | 2.6108 (1.40–4.87) | 0.0025 |  |  |  |
| OR: Odds Ratio. CI: Confidence Interval | | | | | |


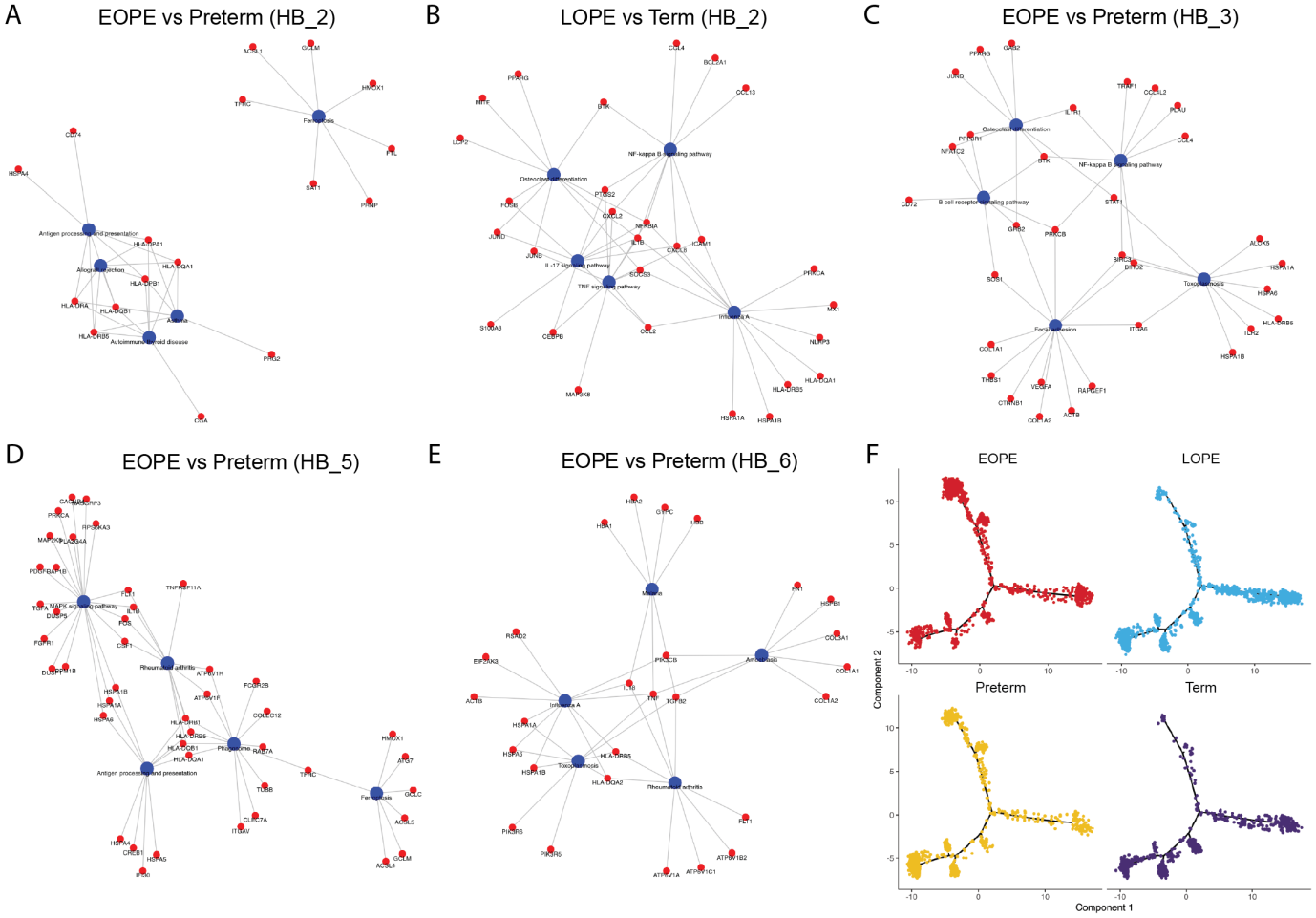


**Supplementary Figure 1**. KEGG pathway enrichment in the most altered Hofbauer cell subclusters between control and preeclampsia groups. (A) KEGG pathway network in the HB_2 subcluster. comparing the EOPE and preterm groups. (B) KEGG pathway network in the HB_2 subcluster. comparing the LOPE and term groups. (C) KEGG pathway network in the HB_3 subcluster. comparing the EOPE and preterm groups. (D) KEGG pathway network in the HB_5 subcluster. comparing the EOPE and preterm groups. (E) KEGG pathway network in the HB_6 subcluster. comparing the EOPE and preterm groups. (F) Inference of pseudotime trajectories for trophoblast cells. color-coded according to groups. EOPE, early-onset preeclampsia; LOPE, late-onset preeclampsia.


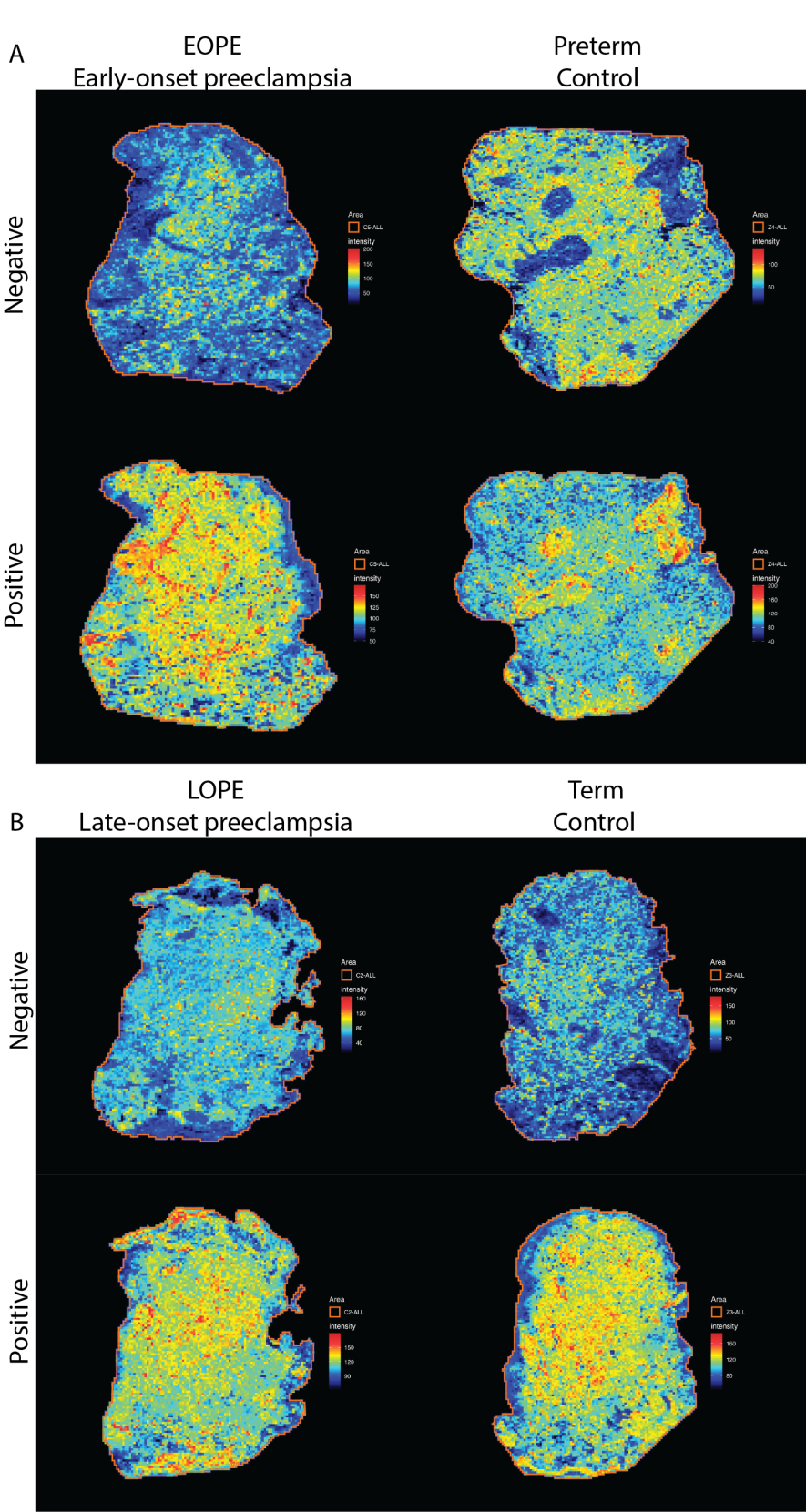


**Supplementary Figure 2**. The total levels of both negative and positive metabolites were assessed through spatial metabolomics analysis. (A) Spatial distribution and heatmap showed the total levels of negative and positive metabolites in EOPE and preterm placentas. (B) Spatial distribution and heatmap showed the total levels of negative and positive metabolites in LOPE and term placentas. EOPE, early-onset preeclampsia; LOPE, late-onset preeclampsia.


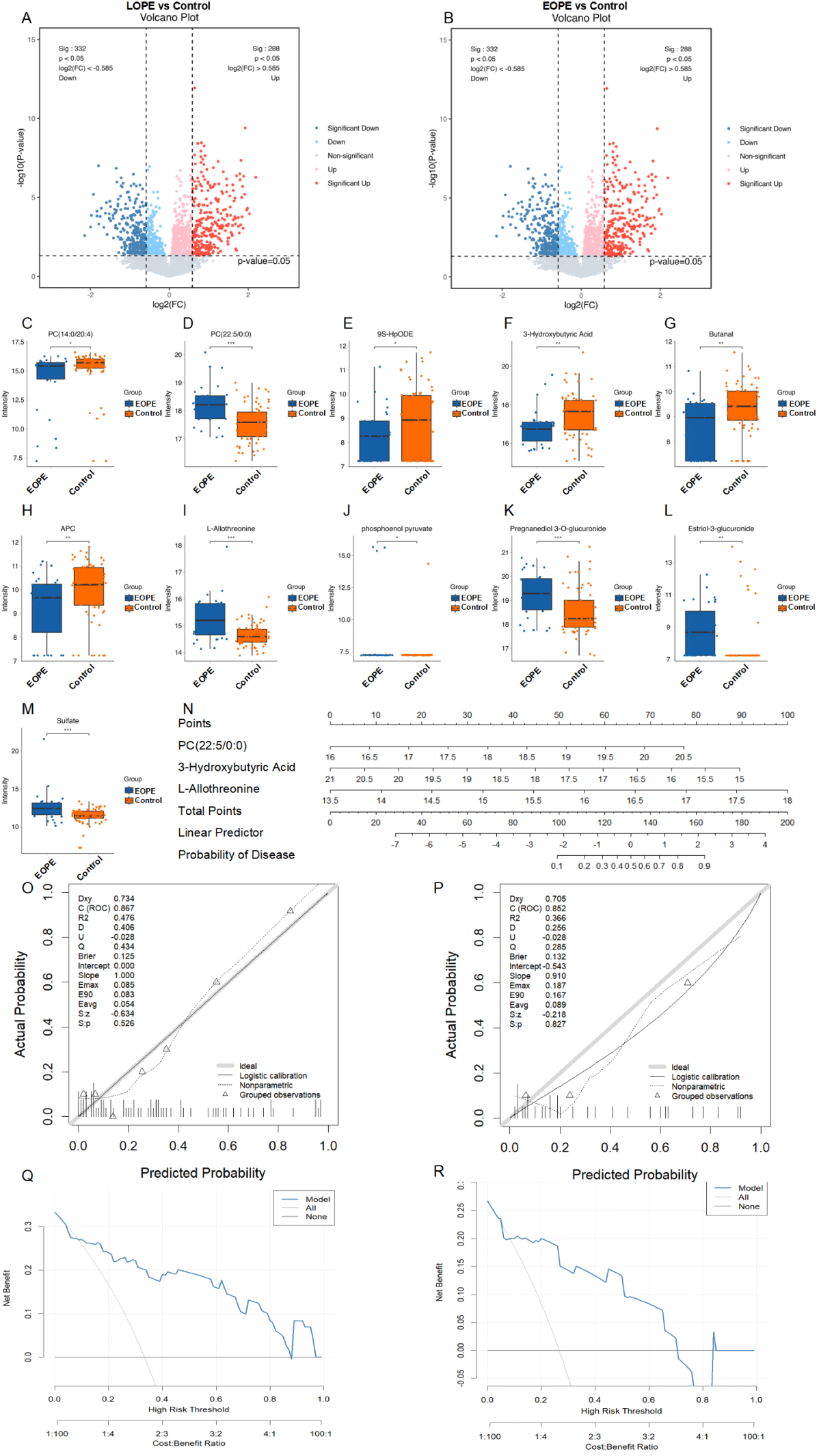


**Supplementary Figure 3**. Serum metabolomic analysis and predictive modeling for early-onset preeclampsia. (A-B) Volcano plots showing differential metabolites in maternal serum for late-onset preeclampsia vs. control (A. LOPE vs. control) and early-onset preeclampsia vs. control (B. EOPE vs. control). Red and blue dots represent significantly upregulated and downregulated metabolites. respectively (|log₂FC| > 0.585. p < 0.05). (C-M) Boxplots showing serum levels of 11 differential metabolites in early-onset preeclampsiaand (blue) gestational age-matched controls(orange) . selected based on KEGG pathway overlap with placental spatial metabolomics. Statistical significance was assessed by Wilcoxon rank-sum test. (N) Nomogram of the predictive model for early-onset preeclampsia constructed using three selected serum metabolites. Total points correspond to individual risk probability. (O-P) Calibration curves of the prediction model in the training (O) and validation (P) sets. The orange line represents the observed prediction. the dashed blue line indicates bias-corrected performance. and the black dashed line represents the ideal prediction. Hosmer-Lemeshow test p-values are shown in each panel.(Q-R) DCA evaluating the net clinical benefit of the prediction model across a range of threshold probabilities in the training set (Q) and validation set (R). In both panels. the red line represents the "treat-all" strategy. the green line represents the "treat-none" strategy. and the blue line represents the prediction model. EOPE, early-onset preeclampsia; LOPE, late-onset preeclampsia. *, P < 0.05; **, P < 0.01; ***, P < 0.001; ****, P < 0.0001; ns, not significant.
